# Assessment of the neuropsychomotor development in the first year of life of premature infants with and without bronchopulmonary dysplasia

**DOI:** 10.5935/0103-507X.20180023

**Published:** 2018

**Authors:** Letycia Vieira Silva, Lúcio Borges de Araújo, Vivian Mara Gonçalves de Oliveira Azevedo

**Affiliations:** 1 Residência Multiprofissional e em Área Profissional da Saúde, Hospital de Clínicas, Faculdade de Medicina, Universidade Federal de Uberlândia - Uberlândia (MG), Brasil.; 2 Faculdade de Matemática, Universidade Federal de Uberlândia - Uberlândia (MG), Brasil.; 3 Faculdade de Educação Física e Fisioterapia, Universidade Federal de Uberlândia - Uberlândia (MG), Brasil.

**Keywords:** Infant, premature, Infant, low birth weight, Bronchopulmonary dysplasia, Developmental disabilities, Risk factors

## Abstract

**Objective:**

To compare the neuropsychomotor development in the first year of life of premature infants with and without bronchopulmonary dysplasia.

**Methods:**

A cross-sectional retrospective study was conducted between January 1, 2014, and December 30, 2015, with premature infants weighing < 1,500g at birth and diagnosed with bronchopulmonary dysplasia at the corrected ages of 6 and 9 months, assessed using the DENVER II Developmental Screening Test. Quantitative variables were described as the means, medians and standard deviations. Variables with normal distribution were tested using Student's *t* test; otherwise, the Mann-Whitney test was used, considering significance at p-value < 0.05. Qualitative variables were expressed as frequencies and percentages. Logistic regression was used with odds ratio analysis to evaluate the effects of other variables as risk factors for changes in neuropsychomotor development.

**Results:**

Infants with bronchopulmonary dysplasia showed greater developmental delay compared with those without bronchopulmonary dysplasia (p-value = 0.001). The factors associated with a higher incidence of changes in neuropsychomotor development, in addition to bronchopulmonary dysplasia, were antenatal steroid, gender, birth weight, 5-minute Apgar score, Score for Neonatal Acute Physiology-Perinatal Extension, duration of oxygen therapy, duration of mechanical ventilation and length of hospital stay. Other variables may also have influenced the result, such as drug use by mothers of infants with bronchopulmonary dysplasia.

**Conclusion:**

Bronchopulmonary dysplasia associated with other pre- and postnatal factors may be considered a risk factor for delayed neuropsychomotor development in the first year of life in premature infants born weighing less than 1,500g.

## INTRODUCTION

Every year, 15 million premature infants are born worldwide, and 1 million die within a few days after birth. Brazil ranks tenth in the list of countries with the highest numbers of premature deliveries.^(^^1^^)^

Consequently, there has been an increase in the occurrence of morbidities, leaving premature infants more susceptible and vulnerable to developmental deficits.^(^^2^^)^ Bronchopulmonary dysplasia (BPD) is the most frequent chronic lung disease in the neonatal period affecting premature infants and contributing to their morbidity and mortality.^(^^3^^)^ The cause of BPD is considered multifactorial and includes prematurity, prolonged exposure to mechanical ventilation (MV) and oxygen therapy, low birth weight and pre- and postnatal events, such as inflammation and infections.^(^^4^^)^ However, some ventilatory strategies have been used as protective measures of MV-induced lung injury in premature infants, which has reduced the incidence of BPD.^(^^5^^)^

BPD has been considered a risk factor for changes in neuropsychomotor development (NPMD), which may manifest early, with significant delays.^(^^6^^)^ The severity of BPD is a predictor of functional, behavioral and sensory deficits.^(^^7^^)^

Newborns with BPD present compromised height and weight development since they have low nutritional intake and increased energy needs,^(^^8^^)^ directly affecting their growth.^(^^7^^)^ Other factors, such as frequent episodes of hypoxia, hypercapnia and respiratory acidosis, may also compromise the central nervous systems of premature infants.^(^^9^^)^

In addition, the time of MV, high concentrations of oxygen,^(^^10^^)^ prolonged hospital stay,^(^^11^^)^ excessive stimuli,^(^^12^^)^ invasive and painful procedures,^(^^13^^)^ the restriction of spontaneous movements^(^^14^^)^ and improper positioning^(^^15^^)^ can contribute to the emergence of delays and, consequently, changes in the NPMD of these children.^(^^6^^)^

Once the hypothesis that BPD damages the NPMD of the premature infants is confirmed, it will be possible to develop strategies and therapeutic interventions to prevent and/or minimize possible sequelae due to prematurity and BPD.

The objective of this study was to compare the NPMD in the first year of life of premature infants born weighing < 1,500g with and without BPD.

## METHODS

A cross-sectional retrospective study was conducted in the medical archive department of a reference university hospital in the state of Minas Gerais, Brazil, from January 1, 2014, to December 30, 2015. The study was approved by the institutional Research Ethics Committee (protocol number: 1.331.951). As the data were collected via analysis of medical records, the informed consent form was not required.

First, the data on the premature infants included in the neonatology database of the hospital were analyzed to select the records according to the inclusion criteria. After the selection, the eligible patients' medical records were consulted. The medical records were evaluated by three researchers.

The records of infants born premature (gestational age below 37 weeks, assessed by the New Ballard score) with birth weights of less than 1,500g and diagnosed with and without BPD, monitored by the high-risk follow-up clinic, who were evaluated for NPMD at the corrected ages of 6 and 9 months using the DENVER II Developmental Screening Test were included. BPD was defined as dependence on oxygen at concentrations above 21% for a period equal to or greater than 28 days.^(^^16^^)^

The NPMD assessment was performed by two properly trained physical therapists from the institution. The choice to evaluate the infants at the corrected gestational ages of 6 and 9 months was made based on the importance of motor milestones at this age and on the clinic's routine protocol.

During the study period, 239 premature infants were born. Of these, 160 were excluded due to death (77), transfer (5), medical records not found in the archive (5), NPMD assessment not found in the medical record (30) and incomplete evaluations at the corrected ages of 6 and 9 months (43). Thus, 79 medical records were evaluated, 40 of infants with BPD and 39 of infants without BPD.

The DENVER II test is a standardized instrument used to screen for children aged zero to six years with risk of global developmental delay. The test consists of 125 items, subdivided into four domains and functions: personal-social, fine motor adaptive, language and gross motor.^(^^17^^)^

The corrected age of the premature infants was used to determine the instrument's score. At the end of the test, the general neuropsychomotor performance was classified according to the items. The collected data were organized into a worksheet in Microsoft Office Excel^®^ 2010.

The variables studied were divided into maternal and neonatal characteristics, which were subdivided into quantitative variables (maternal age, gestational age, birth weight, 5-minute Apgar score, duration of oxygen therapy, duration of MV, length of hospital stay and Score for Neonatal Acute Physiology-Perinatal Extension - SNAP-PE score) and qualitative variables (children of drug-using mothers, maternal education, receipt of prenatal care, chorioamnionitis, antenatal steroid use, newborn gender, surfactant use, neonatal infection, peri-intraventricular hemorrhage and leukomalacia).

The quantitative variables within each group were described using means, medians and standard deviations. In addition, the Shapiro-Wilk normality test was applied. For variables with normal distribution in the two groups, Student's *t* test was used to compare groups; otherwise, the Mann-Whitney test was used, considering significance at p-value < 0.05.^(^^18^^)^ The qualitative variables were described (frequencies and percentages) using double-entry tables.

Logistic regression with odds ratio analysis was used to evaluate the effects of the other variables as risk factors for changes in NPMD.

## RESULTS

The study included 79 charts of very-low-birth-weight premature infants with and without BPD. For all infants, the qualitative and quantitative variables and the DENVER II test were analyzed at the corrected ages of 6 (n = 40, 21 dysplastic and 19 non-dysplastic infants) and 9 (n = 39, 19 dysplastic and 20 non-dysplastic infants) months.

There were significant differences in maternal and neonatal characteristics between the groups with and without BPD. The comparisons of the variables are summarized in table 1.

**Table 1:** Comparison of the studied groups in relation to maternal and neonatal characteristics

Characteristics	Group 1 N = 40	Group 2 N = 39	p value
Maternal characteristics			
Age (years)	25.00 (20 - 30)	27.00 (23 - 30)	0.251
Schooling (years of study)			0.080
1-7	9 (22.5)	2 (5.1)	
8-11	25 (62.5)	26 (66.7)	
12 or more	6 (15)	11 (28.2)	
Use of drugs	13 (32.5)	5 (12.8)	0.034
Receipt of prenatal care	37 (92.5)	38 (97.4)	0.306
Antenatal use of steroids	26 (65)	37 (69.2)	0,689
Congenital infection	2 (5)	3 (7.7)	0.622
Chorioamnionitis	9 (22.5)	6 (15.4)	0.418
Neonatal characteristics			
Sex			0.191
Female	18 (45)	12 (30.8)	
Male	22 (55)	27 (69.2)	
Gestational age (weeks)	28.00 (26 - 30)	30.00 (29 - 32)	0.002
Birth weight (grams)	922.23 ± 229.91	1,236.05 ± 209.34	0.000
5-minute Apgar score	7.00 (7 - 8)	9.00 (8 - 9)	0.000
SNAP-PE	31.50 (24 - 53)	20.00 (12 - 40)	0.002
Use of surfactant	26 (65)	12 (30.8)	0.002
Duration of oxygen therapy (days)	61.00 (47.25 - 77)	3.00 (1 - 5)	0.000
Duration of mechanical ventilation (days)	6.50 (3 - 26.25)	0.00 (0 - 1)	0.000
Infection	20 (50)	9 (23.1)	0.012
Peri-intraventricular hemorrhage (grade)			0.019
0	24 (60)	33 (84.6)	
1	7 (17.5)	4 (10.3)	
2	5 (12.5)	0 (0)	
3	4 (10)	5 (2.1)	
Leukomalacia	0 (0)	1 (2.6)	0.232
Length of hospital stay	84.00 (73.50 - 104.50)	50.00 (41 - 57)	0.000

SNAP-PE - Score for Neonatal Acute Physiology with Perinatal Extension. Group 1 - premature infants with bronchopulmonary dysplasia; Group 2 - premature infants without bronchopulmonary dysplasia. For the qualitative variables, the results are expressed as n (%); for the quantitative variables, the results are expressed as the means ± standard deviations for parametric tests and medians (interquartile ranges) for the nonparametric tests.

When comparing the performance classification of the two groups of infants as to NPMD by the corrected age, a significant difference between the groups was observed (p = 0.001), indicating that premature infants with BPD had a longer delay in NPMD compared with those without BPD. In addition, a significantly greater number of failures in the personal-social domain in the group of premature infants with BPD was observed (p = 0.001). The NPMD results are presented in table 2.

**Table 2:** Comparison of neuropsychomotor development between the groups studied, according to the DENVER II Developmental Screening Test

Neuropsychomotor development	Group 1 N = 40 n (%)	Group 2 N = 39 n (%)	Total N = 79 n (%)	p value
Personal-social				0.001
Normal	26 (65)	35 (89.7)	61 (77.2)	
1 failure	6 (15)	2 (5.1)	8 (10.1)	
2 failures	4 (10)	0 (0)	4 (5.1)	
3 failures	0 (0)	2 (5.1)	2 (2.5)	
1 caution	4 (10)	0 (0)	4 (5.1)	
Language				0.284
Normal	28 (70)	33 (84.6)	61 (77.2)	
1 failure	8 (20)	6 (15.4)	14 (17.7)	
2 failures	1 (2.5)	0 (0)	1 (1.3)	
1 caution	1 (2.5)	0 (0)	1 (1.3)	
2 cautions	1 (2.5)	0 (0)	1 (1.3)	
3 cautions	1 (2.5)	0 (0)	1 (1.3)	
Fine and adaptive motor				0.121
Normal	29 (72.5)	35 (89.7)	64 (81)	
1 failure	4 (10)	1 (2.6)	5 (6.3)	
2 failures	2 (5)	1 (2.6)	3 (3.8)	
3 failures	2 (5)	0 (0)	2 (2.5)	
1 caution	1 (2.5)	2 (5)	3 (3.8)	
2 cautions	2 (5)	0 (0)	2 (2.5)	
Gross motor				0.410
Normal	16 (39)	21 (52.5)	37 (45.7)	
1 failure	14 (34.1)	11 (27.5)	25 (30.9)	
2 failures	3 (7.3)	3 (7.5)	6 (7.4)	
3 failures	1 (2.4)	3 (7.5)	4 (4.9)	
1 caution	3 (7.3)	1 (2.5)	4 (4.9)	
2 cautions	4 (9.8)	1 (2.5)	5 (6.2)	
Performance classification				0.001
Not normal	20 (50)	5 (12.8)	25 (31.6)	
Suspect	10 (25)	14 (35.9)	24 (30.4)	
Normal	10 (25)	20 (51.3)	30 (38)	

Group 1 - infants with bronchopulmonary dysplasia; Group 2 - infants without bronchopulmonary dysplasia.

Logistic regression was performed to identify the factors associated with a higher incidence of changes in NPMD (Table 3). Analysis of the effect of each variable separately (univariate analysis) revealed that seven variables related to a highest probability of changes in NPMD (antenatal steroid use, birth weight, SNAP-PE score, duration of oxygen therapy, duration of MV, length of hospital stay, BPD). In the joint analysis (multivariate analysis), six variables were identified (antenatal steroid use; sex, with female considered a protective factor; birth weight; 5-minute Apgar score; SNAP-PE score; duration of oxygen therapy).

**Table 3:** Factors associated with changes in neuropsychomotor development in infants with and without bronchopulmonary dysplasia

Characteristics	OR (95%CI) Univariate analysis	p value	OR (95%CI) Multivariate analysis	p value
Maternal				
Age (years)	0.99 (0.92 - 1.06)	0.857	-	-
Education	0.74 (0.35 - 1.56)	0.436	-	-
Prenatal	0.52 (0.05 - 5.32)	0.589	-	-
Congenital infection	0.91 (0.14 - 5.80)	0.923	-	-
Chorioamnionitis	1.88 (0.53 - 6.55)	0.321	-	-
Antenatal steroid use	0.26 (0.08 - 0.81)	0.020	0.26 (0.08 - 0.83)	0.023
Neonatal				
Sex	0.44 (0.17 - 1.12)	0.088	0.11 (0.02 - 0.52)	0.005
Birth weight	0.99 (0.99 - 0.99)	0.000	0.99 (0.99 - 0.99)	0.014
5-minute Apgar score	0.84 (0.58 - 1.23)	0.379	2.10 (1.07 - 4.10)	0.029
SNAP-PE	1.04 (1.01 - 1.07)	0.008	1.04 (1.00 - 1.09)	0.035
Surfactant use	2.11 (0.83 - 5.38)	0.114	-	-
Oxygen therapy duration	1.03 (1.01 - 1.04)	0.001	1.06 (1.01 - 1.11)	0.013
MV duration	1.16 (1.03 - 1.31)	0.014	-	-
Infection	2.67 (0.96 - 7.39)	0.058	-	-
Bleeding	1.53 (0.85 - 2.76)	0.152	-	-
Leukomalacia	1.00 (-)	0.999	-	-
Length of hospital stay (days)	1.03 (1.01 - 1.05)	0.001	-	-
BPD	0.29 (0.11 - 0.75)	0.011	7.27 (0.84 - 62.43)	0.070

OR - Odds ratio; 95% CI - 95% confidence interval; SNAP-PE: Score for Neonatal Acute Physiology with Perinatal Extension; MV - mechanical ventilation; BPD - bronchopulmonary dysplasia.

## DISCUSSION

BPD alone does not represent a risk factor for NPMD delay in premature infants born weighing <1,500g. Other variables, such as antenatal steroid use, sex, birth weight, 5-minute Apgar score, SNAP-PE score, duration of oxygen therapy, duration of MV and length of hospital stay, when combined with BPD, increase the chances of NPMD delay.

Corroborating with our findings, Holditch-Davis et al.^(^^18^^)^ concluded that NPMD delay is not a consequence of BPD alone; these changes are likely consequences of prolonged hospitalizations, duration of MV, nutritional compromises, lack of opportunities for interaction and inadequate learning and stimulation.

Martins et al.^(^^19^^)^ found that 90% of premature infants born with low weight and who developed BPD exhibited changes in motor development, axial hypotonia and hypertonia of the lower limbs, according to the Bayley Scale of Infant Development. Oliveira et al.^(^^20^^)^ also found a significant association with low birth weight and BPD. Children with BPD were four times more likely to exhibit changes in motor development before 6 months of corrected gestational age.

In a study conducted in Australia,^(^^21^^)^ premature children were more vulnerable to cognitive, educational and behavioral deficits, with BPD being an additional risk factor that exacerbated these deficits. However, BPD does not appear to be associated with a specific neuropsychological impairment, but with a global impairment. For example, children with BPD exhibit changes in tone, hearing, speech and gross motor skills, such as rolling, crawling and walking.^(^^22^^)^ Deficits in language, reading, attention and fine motor skills were also observed in children with low birth weight who underwent MV and prolonged use of oxygen with evolution to BPD.^(^^23^^)^

An opposite result was found by Robertson et al.,^(^^24^^)^ who found similar physical, psychoeducational and school performance levels between children with and without BPD who received supplemental oxygen, with the exception of the intelligence quotient (IQ), which was lower in children who received supplemental oxygen for longer. However, throughout life, children with BPD can recover from potential delays. Trittmann et al.^(^^25^^)^ also observed no difference in the Bayley Scale of Infant Development III composite score (cognitive, communication and motor) at 18 months of corrected age in children with BPD.

Similar to BPD, low birth weight also increased the risk of NPMD delay. In addition, other variables associated with prematurity were found to be determinants of changes in NPMD. Similar results were found by Amador et al.^(^^8^^)^ and Koyama et al.,^(^^22^^)^ who, when analyzing the NPMD of children with and without BPD, observed that the 5-minute Apgar score, prolonged oxygen use, MV and length of hospital stay were factors associated with development of BPD and changes in NPMD.^(^^8^^)^ Landry et al.^(^^26^^)^ also found a significant association between NPMD delay and the variables birth weight, length of hospital stay and duration of MV, in addition to other factors such as BPD severity, neurological impairment, neonatal seizures and ischemic hypoxic encephalopathy. Therefore, the authors found that children with BPD had a higher risk of developing cerebral palsy and delays in cognitive and motor functions.

In our study, cerebral hemorrhage was another obvious risk factor for NPMD delay, although we did not find a significant association in the logistic regression. Corroborating our results, Martins et al.^(^^19^^)^ also did not observe a significant association between cerebral hemorrhage and changes in NPMD but found a risk of 1.7 for changes in NPMD in the group with BPD and cerebral hemorrhage.

Regarding the analysis of the maternal variables associated with NPMD, children of drug-using mothers had higher incidence rates of BPD and NPMD delay. These results refute those found by Gasparin et al.,^(^^27^^)^ who analyzed the development of 25 premature and full-term children of drug-using mothers using the Test of Infant Motor Performance (TIMP) scale and did not identify delays. However, they observed that weights at birth and at the time of the evaluation were lower than those of the children of non-users, suggesting that drug use is a risk factor for prematurity and low birth weight.

For the other maternal variables analyzed (maternal age, maternal schooling, receipt of prenatal care, infection and antenatal steroid use), no significant differences were found between the groups. Cunha et al.^(^^28^^)^ and Lima et al.^(^^29^^)^ also did not observe any influences of these variables on the incidence of BPD. However, some of these factors may induce premature birth and increase the risk of delayed NPMD. Thus, proper prenatal care is extremely important.^(^^29^^)^ In the logistic regression analysis, the antenatal use of steroids was shown to be a protective factor for changes in NPMD, likely because it accelerates fetal pulmonary maturation when there is a risk of premature delivery, thus reducing the incidence of respiratory diseases and dependence on ventilatory support.

The hypotheses raised regarding the influence of BPD on NPMD are diverse. The observed changes may be related to episodes of hypoxia,^(^^19^^,^^20^^)^ frequent hospital readmissions, nutritional deficits^(^^5^^)^ and other complications, such as intraventricular hemorrhage or periventricular leukomalacia, which may be determinant for the occurrence of delays.^(^^18^^)^ It should be noted that the two groups, with and without BPD, were made up of premature infants born weighing <1,500 g, all of whom were at risk of delayed NPMD due to prematurity.

One of the limiting factors of the study was the absence of the Denver II Test result in some medical records, in addition to it being considered a screening test rather than a diagnostic test for delays. However, there are few scales validated for the Brazilian infant population feasible to be applied in follow-up outpatient clinics.

## CONCLUSION

Bronchopulmonary dysplasia combined with other pre- and postnatal factors may be considered a risk factor for delayed neuropsychomotor development in the first year of life in premature infants born weighing < 1,500g. These results reinforce the importance of a multiprofessional approach in the follow-up of these infants in the first years of life to identify possible delays and refer them to early intervention, reducing the risks of inadequate growth and development.

It is expected that the results of this study will serve as support for a possible redirection toward more effective strategies, such as preventive measures, intervention and early stimulation, to prevent and/or minimize possible deficits due to prematurity and bronchopulmonary dysplasia.
